# Goal-Directed Intraoperative Fluid Therapy Benefits Patients Undergoing Major Gynecologic Oncology Surgery: A Controlled Before-and-After Study

**DOI:** 10.3389/fonc.2022.833273

**Published:** 2022-04-06

**Authors:** Jiawen Yu, Lu Che, Afang Zhu, Li Xu, Yuguang Huang

**Affiliations:** Department of Anesthesiology, Peking Union Medical College Hospital, Chinese Academy of Medical Sciences and Peking Union Medical College, Beijing, China

**Keywords:** gynecologic oncology, goal-directed fluid therapy, postoperative complication, surgical site infection, hemodynamics

## Abstract

**Background:**

Fluid management during major gynecologic oncology surgeries faces great challenges due to the distinctive characteristics of patients with gynecologic malignancies as well as features of the surgical procedure. Intraoperative goal-directed fluid therapy (GDFT) has been proven to be effective in reducing postoperative complications among major colorectal surgeries; however, the efficacy of GDFT has not been fully studied in gynecologic malignancy surgeries. This study aimed to discuss the influence of GDFT practice in patients undergoing major gynecologic oncology surgery.

**Methods:**

This study was a controlled before-and-after study. From June 2015 to June 2018 in Peking Union Medical College Hospital, a total of 300 patients scheduled for elective laparotomy of gynecological malignancies were enrolled and chronologically allocated into two groups, with the earlier 150 patients in the control group and the latter 150 patients in the GDFT group. The GDFT protocol was applied by Vigileo/FloTrac monitoring of stroke volume and fluid responsiveness to guide intraoperative fluid infusion and the use of vasoactive agents. The primary outcome was postoperative complications within 30 days after surgery. The secondary outcome included length of stay and time of functional recovery.

**Results:**

A total of 249 patients undergoing major gynecologic oncology surgery were analyzed in the study, with 129 in the control group and 120 patients in the GDFT group. Patients in the GDFT group had higher ASA classifications and more baseline comorbidities. GDFT patients received significantly less fluid infusion than the control group (15.8 vs. 17.9 ml/kg/h), while fluid loss was similar (6.9 vs. 7.1 ml/kg/h). GDFT was associated with decreased risk of postoperative complications (OR = 0.572, 95% CI 0.343 to 0.953, *P* = 0.032), especially surgical site infections (OR = 0.127, 95% CI 0.003 to 0.971, *P* = 0.037). The postoperative bowel function recovery and length of hospital stay were not significantly different between the two groups.

**Conclusion:**

Goal-directed intraoperative fluid therapy is associated with fewer postoperative complications in patients undergoing major gynecologic oncology surgery.

## Introduction

Intraoperative fluid management among patients with gynecologic malignancy often meets great challenges due to the unique characteristics of these patients. Firstly, these patients often need to experience multiple courses of chemotherapy both before and after surgery, making them susceptible to malnutrition and anemia. Secondly, routine preoperative fasting and bowel preparation may result in inadequate intravascular volume and is associated with discomfort and poor perioperative outcome. On the other hand, major gynecological surgeries, for instance, comprehensive staging surgeries and cytoreductive surgeries, usually require open laparotomy involving extensive surgical trauma, during which there might be a great loss of fluid including blood, ascites, and invisible hydration through vaporization and respiration.

For better intraoperative fluid management for patients undergoing major gynecologic oncology surgery, the answer might lie in goal-directed fluid therapy (GDFT). Aiming at an adequate cardiac output, GDFT can provide patients with adequate volume and tissue perfusion theoretically, thus resulting in a better postoperative outcome. In other practices, GDFT showed benefits by lowering the risks of postoperative complications compared with conventional free fluid therapy in colorectal surgical patients (1). However, current evidence is sparse concerning gynecologic oncology surgeries. Although it has been recommended in enhanced recovery after surgery (ERAS) protocol for gynecological surgery (2), the efficacy of intraoperative GDFT has been rarely discussed specifically. Moreover, fluid management was poorly followed in clinical practice (3). One cohort study found that ERAS patients received lesser intraoperative fluid administration than control patients; however, the clinical significance needed to be further clarified (4). Therefore, more clinical studies are needed to guide clinical practice. In this study, we conducted a controlled before-and-after study to examine the influence of intraoperative GDFT on postoperative outcome in patients undergoing major gynecologic oncology surgery.

## Methods

### Study Design

This study was a controlled before-and-after study. Patients were recruited from June 2015 to June 2018. The study protocol was approved by the Research Ethics Committee of Peking Union Medical College Hospital on October 21, 2014 (reference number: S-737) and prospectively registered at clinicaltrials.gov (NCT02470221). Written informed consents were obtained from all participants.

### Participants

Patients who met all the following inclusion criteria were considered for recruitment: 1) patients receiving open cytoreductive surgery, radical hysterectomy, and staging surgery of endometrial cancer; 2) age ≥18 years; 3) American Society of Anesthesiologists Physical Status (ASA-PS) classification I–IV; 4) undergoing general anesthesia; and 5) requiring monitoring of direct blood pressure due to comorbidities of patients or the nature of surgical procedures. Patients who met any of the following exclusion criteria will be excluded: 1) emergent surgeries; 2) patients with aortic stenosis, peripheral arterial diseases, or other contraindications of arterial canalization; patients with aortic regurgitation; patients with current arrhythmia; and 3) patients who cannot cooperate or refuse to sign a consent.

### Anesthesia Management

All patients accepted general anesthesia induced by propofol, fentanyl, and rocuronium and maintained under target-controlled infusion (TCI) of propofol (plasma concentration 3–5 μg/ml). For airway management, all participants received endotracheal intubation and were under volume control mode with a tidal volume of 8 ml/kg.

### Intraoperative GDFT Management

**Figure 1:**
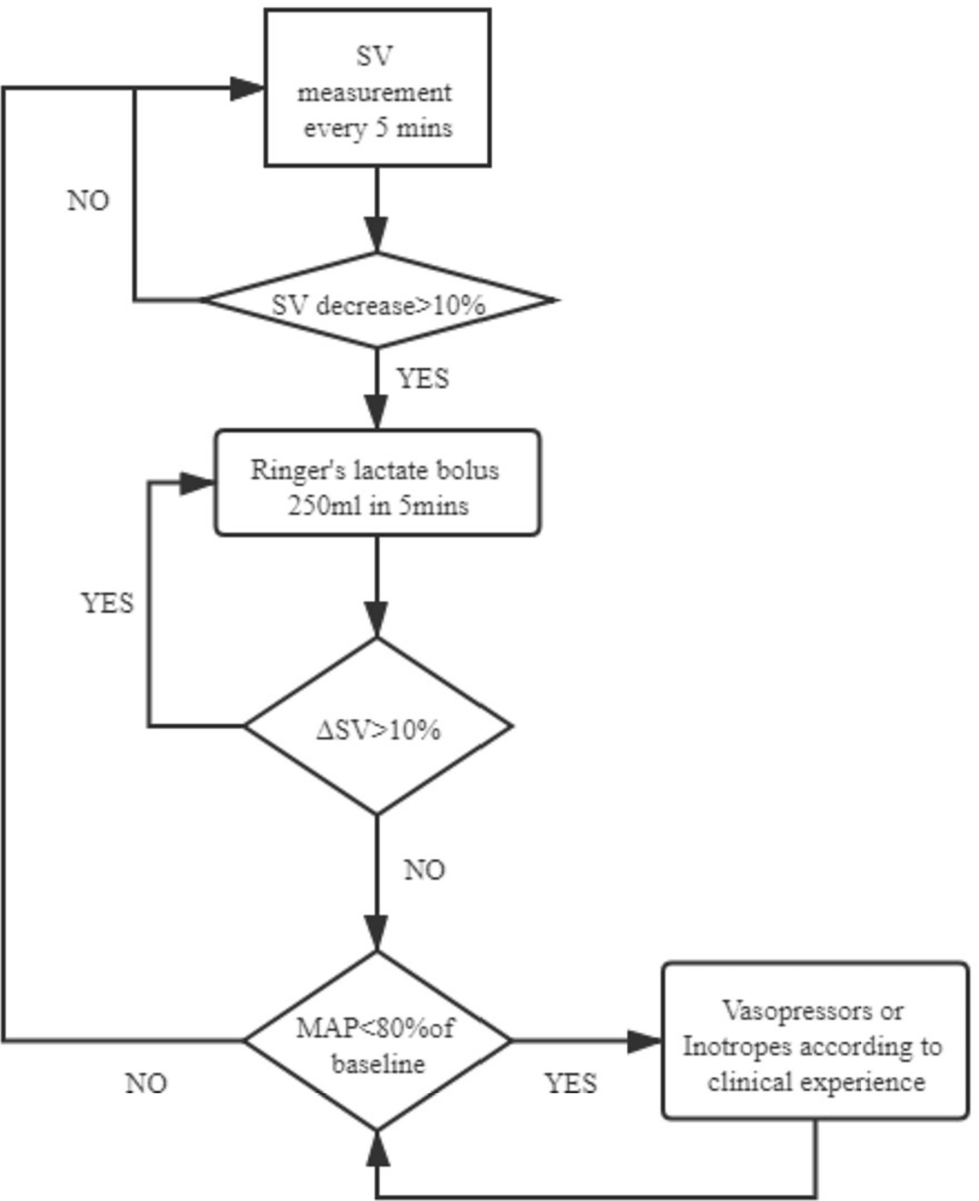
GDFT protocol. GDFT, goal-directed fluid therapy; SV, stroke volume; MAP, mean arterial pressure.

After the patients’ entry to the operation room and before anesthesia induction, a maintenance Ringer’s lactate infusion at 3 ml/kg/h was performed in both groups. A 20-G radial arterial line was used for continuous arterial pressure monitoring in both the GDFT group and the control group, while in the GDFT group, the patient’s arterial line was connected to the Vigileo/FloTrac system (Edwards Lifesciences, Irvine, CA, USA) to calculate stroke volume (SV). In the GDFT group, SV was recorded every 5 min and a 3-ml/kg Ringer’s lactate bolus was administered over 5 min to assess fluid responsiveness. An increase in SV >10% was considered as fluid responders and further administered with another bolus for reassessment. For non-responders, if the patient experienced hypotension (mean arterial pressure < 80% of baseline), vasopressors were used. The intraoperative GDFT protocol is summarized in **Figure 1**. For the control group, conventional fluid therapy was applied according to the anesthesiologists’ experience, based on the principle of crystalloid vs. colloid solution = 2–3:1 and total volume of fluid adjusted in accordance with fasting time and the patients’ weight, heart rate, blood pressure, and urine output.

### Outcome

The primary outcome of the study was composite postoperative complications within 30 days after surgery, including cardiac events (severe arrhythmia, hypotension, hypertension), infectious complications (surgical site infection, pneumonia, urinary tract infection, bacteremia), gastrointestinal complications (ileus, diarrhea, postoperative nausea, and vomiting), hematologic complications (deep venous thrombosis, pulmonary embolism, massive postoperative red blood cell transfusion), acute kidney injury, anastomotic fistula, and lymphocele. The severity of postoperative complications was classified according to the Clavien–Dindo classification (5). The secondary outcomes were length of hospital stay, requirement for intensive care, and time of first exhaust, defecation, urination, and first oral intake of liquid or semi-liquid as indicators for functional recovery. Postoperative follow-up was performed by investigators *via* daily ward visits and telephone follow-up after discharge, supplemented by patients’ medical records.

### Sample Size and Statistical Analysis

The previously observed incidence of postoperative complications in our institute was 60%, and a power analysis indicated a sample size of 150 patients in each group required for a reduction from 60% to 45% (relative 25% reduction), with a power of 0.8 and type 1 error (*α*) = 0.05. Independent Student’s *t*-test, chi-square test, Fisher’s exact test, or Mann–Whitney *U* test was used for comparison of baseline, intraoperative, and postoperative information between the control and GDFT group. Multivariable logistic analysis was used to estimate the association between primary outcome (patients experienced one or more postoperative complications) and intraoperative fluid management method adjusting for age, ASA-PS classification, history of chemotherapy, and prolonged operations (surgical time > 4 h) primarily based on clinical concerns. Statistical analysis was performed on SPSS Statistics 22.0 (IBM Corp., USA) and R version 3.6.3 (R Foundation for Statistical Computing, Vienna, Austria). A *P*-value (two-sided) <0.05 was considered statistically significant.

## Results

**Figure 2:**
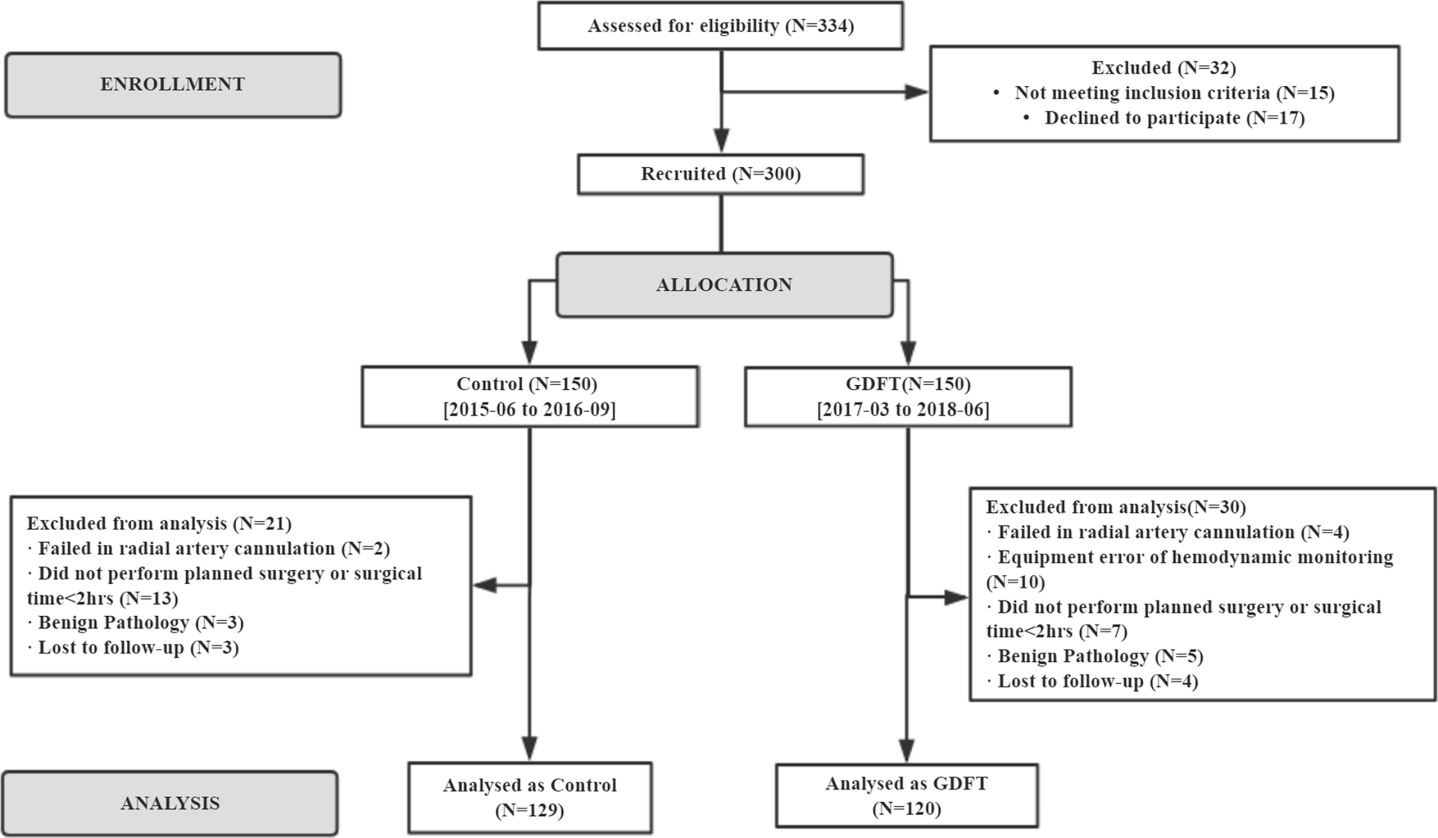
CONSORT flow diagram.

A total of 300 patients were enrolled in the study between June 2015 and June 2018. From June 2015 to September 2016, 150 patients were included in the control group. Starting from March 2017 to June 2018, another 150 patients were allocated to the GDFT group. These patients were further selected into study analysis according to the actual operation performed and postoperative pathology results. Two hundred and forty-nine patients receiving elective open cytoreductive surgery for gynecological cancer were included in the final analysis, with 129 in the control group and 120 in the GDFT group (**Figure 2**). Patients’ baseline characteristics including age, body mass index (BMI), baseline hemoglobin, and creatinine were not significantly different between the two groups. However, the ASA-PS classification was significantly higher in the GDFT group with 27.5% of patients classified to ASA-PS III, while there were only 5.4% in the control group (*P* < 0.001). Significantly more patients in the GDFT group had a history of hypertension (29.2% vs. 17.1%, *P* = 0.023) or cerebral infarction (6.0% vs. 0.0%, *P* = 0.012) than in the control group (**Table 1**).

**Table 1:** Patients’ baseline characteristics.

	Control (N = 129)	GDFT (N = 120)	P-value
*Age (years, mean ± SD)*	53.2 ± 12.7	54.9 ± 10.9	0.264^a^
*BMI (kg/m^2^, mean ± SD)*	22.8 ± 3.3	23.3 ± 3.4	0.283^a^
*Baseline MAP (mmHg, mean ± SD)*	91.7 ± 12.3	89.7 ± 11.2	0.167^a^
*Baseline* h*emoglobin (g/L, mean ± SD)*	121.1 ± 15.2	121.9 ± 14.4	0.664^a^
*Baseline* c*reatinine (*μ*mol/L, mean ± SD)*	58.6 ± 11.8	59.6 ± 13.0	0.530^a^
*ASA-PS classification ≥III (n, %)*	7 (5.4%)	33 (27.5%)	<0.001^b^
*Medical* h*istory (n, %)*			
* Hypertension*	22 (17.1%)	35 (29.2%)	0.023^b^
* Diabetes mellitus*	19 (14.7%)	14 (11.7%)	0.476^b^
* Coronary artery disease*	6 (4.7%)	2 (1.7%)	0.284^c^
* Cerebral infarction*	0 (0.0%)	6 (6.0%)	0.012^c^
* Tumor*	9 (7.0%)	16 (13.3%)	0.095^b^
*History of* c*hemotherapy*	83 (64.3%)	79 (65.8%)	0.805^b^
*History of* r*adiotherapy*	5 (3.2%)	2 (1.7%)	0.449^c^
*History of* s*urgery*	37 (28.7%)	23 (19.2%)	0.079^b^

BMI, body mass index; MAP, mean arterial pressure; ASA-PS, American Society of Anesthesiologists Physical Status; GDFT, goal-directed fluid therapy.

a Student’s t-test.

b Chi-square test.

c Fisher’s exact test.

In terms of intraoperative management (**Table 2**), the operation time showed no significant difference between the two groups. Patients in the control group received more crystalloid than patients in the GDFT group (12.4 ± 4.1 vs. 10.5 ± 3.5 ml/kg/h, *P* < 0.001), while there was no difference regarding infusion of colloids (3.5 ± 2.3 vs. 3.2 ± 1.8 ml/kg/h, *P* = 0.197). For total intraoperative fluid balance, patients in the GDFT group had a total fluid balance of 8.9 ± 4.9 ml/kg/h, while it was 10.8 ± 6.5 ml/kg/h for the control group. The fluid loss used in the calculation included urine, blood loss, and ascites if any, while no invisible fluid loss was calculated considering its difficulty to quantify. There was no significant difference in total fluid output between the two groups (7.1 ± 5.4 vs. 6.9 ± 4.8 ml/kg/h, *P* = 0.691). In terms of the implementation of vasoactive medications, generally, more vasopressors were used in the GDFT group and significantly more patients in the GDFT group received continuous phenylephrine infusion.

**Table 2:** Intraoperative information.

	Control (N = 129)	GDFT (N = 120)	P-value
*Operation* t*ime* [*min, median* (*IQR)*]	233 (195 to 290)	248 (211 to 305)	0.241^a^
*Fluid* b*alance (ml/kg/h, mean ± SD)*	10.8 ± 6.5	8.9 ± 4.9	0.011^b^
* Total* i*nfusion (ml/kg/h, mean ± SD)*	17.9 ± 7.0	15.8 ± 6.3	0.013^b^
* Crystalloid infusion (ml/kg/h, mean ± SD)*	12.4 ± 4.1	10.5 ± 3.5	<0.001^b^
* Colloid* i*nfusion (ml/kg/h, mean ± SD)*	3.5 ± 2.3	3.2 ± 1.8	0.197^b^
* Total* o*utput (ml/kg/h, mean ± SD)*	7.1 ± 5.4	6.9 ± 4.8	0.691^b^
* Urine (ml/kg/h, mean ± SD)*	3.3 ± 1.8	2.9 ± 1.6	0.095^b^
* Estimated* blood l*oss* [*ml, median* (*IQR)*]	475 (225 to 1,000)	500 (300 to 1,000)	0.398^a^
*Intraoperative* blood t*ransfusion (n, %)*	59 (45.7%)	56 (46.7%)	0.883^c^
*Bolus of* e*phedrine (n, %)*	41 (31.8%)	50 (41.7%)	0.106^c^
*Bolus of* p*henylephrine (n, %)*	17 (13.2%)	19 (15.8%)	0.552^c^
*Continuous* infusion of p*henylephrine (n, %)*	18 (14.0%)	44 (36.7%)	<0.001^c^
*Presence of ascites*	9 (7.0%)	17 (14.2%)	0.064^c^
*Patients undergoing intestinal anastomosis (n, %)*	20 (15.5%)	19 (15.8%)	0.943^c^

a Mann–Whitney U test.

b Student’s t-test.

c Chi-square test.

More patients experienced postoperative complications in the control group than in the GDFT group (65.9% vs. 52.5%, OR = 0.572, 95% CI 0.343 to 0.953, *P* = 0.032), among which the incidence of surgical site infection was significantly higher in the control group than that in the GDFT group (OR = 0.127, 95% CI 0.003 to 0.971, *P* = 0.037). Other complications including cardiovascular events, infections, gastrointestinal complications, hematologic complications, and other miscellaneous complications showed no differences between the two groups (**Table 3**). In patients undergoing intestinal anastomosis, the incidence of anastomotic fistula was higher in the control group, yet with no statistical significance (2 in 20 vs. 0 in 19, *P* = 0.487). Multivariable analysis was performed to examine the association between GDFT and composite postoperative complications (**Table 4** and **Appendix Table S2**). After adjusting for age, ASA-PS classification, history of chemotherapy, and surgical time, GDFT implementation was strongly associated with reduced risk of postoperative complications in patients who underwent major gynecologic oncology surgery (OR = 0.421, 95% CI 0.241 to 0.733, *P* = 0.002).

**Table 3:** Postoperative complications within 30 days after surgery.

		Control (N = 129)	GDFT (N = 120)	P-value
*Patients with one or more complications, n (%)*		85 (65.9%)	63 (52.5%)	0.032^a^
*Clavien*–*Dindo* c*lassification*	I	61 (47.3%)	47 (39.2%)	0.033^b^
	II	22 (17.1%)	15 (12.5%)	
	IIIa	1 (0.8%)	1 (0.8%)	
	IIIb	1 (0.8%)	0 (0.0%)	
	IVa	0 (0.0%)	0 (0.0%)	
	IVb	0 (0.0%)	0 (0.0%)	
	V	0 (0.0%)	0 (0.0%)	
*Specific* postoperative c*omplications, n (%)*				
*Infectious* c*omplications*				
* Surgical* site i*nfection (SSI)* * Pneumonia*		8 (6.2%) 0 (0.0%)	1 (0.8%) 3 (2.5%)	0.037^c^ 0.110^c^
* Urinary* tract i*nfection*		6 (4.7%)	4 (3.3%)	0.751^c^
* Bacteremia*		1 (0.8%)	0 (0.0%)	1.000^c^
			
*Cardiovascular* c*omplications*				
* Arrhythmia*		2 (1.6%)	1 (0.8%)	1.000^c^
* Hypotension*		2 (1.6%)	0 (0.0%)	0.499^c^
* Myocardial* i*schemia*		1 (0.8%)	0 (0.0%)	1.000^c^
*Gastrointestinal* c*omplications*				
* Ileus*		3 (2.3%)	4 (3.3%)	0.714^c^
* Diarrhea*		11 (8.5%)	6 (5.0%)	0.270^a^
* PONV*		62 (48.1%)	50 (41.7%)	0.311^a^
*Anastomotic fistula^d^*		2 in 20 (10.0%)	0 in 19 (0.0%)	0.487^c^
*Hematologic* c*omplications*				
* Pulmonary* e*mbolism*		1 (0.8%)	1 (0.8%)	1.000^c^
* Deep* venous t*hrombosis*		2 (1.6%)	1 (0.8%)	1.000^c^
* Postoperative RBC* t*ransfusion ≥4* *U*		7 (5.4%)	6 (5.0%)	0.880^a^
*Others*				
* AKI*		3 (2.3%)	3 (2.5%)	1.000^c^
* Lymphocele*		4 (3.1%)	0 (0.0%)	0.123^c^

SSI, surgical site infection; PONV, postoperative nausea and vomiting; RBC, red blood cell; AKI, acute kidney injury.

a Chi-square test.

b Mann–Whitney U test

c Fisher’s exact test.

d Percentages are calculated in patients with intestinal anastomosis.

**Table 4:** Multivariable logistic regression analysis of postoperative complications.

Model	Variables	*Odds ratio (95% CI)*	*P*-*value*
*Crude*	GDFT	0.572 (0.343 to 0.953)	0.032
*Adjusted*	GDFT	0.421 (0.241 to 0.733)	0.002
	Age	1.005 (0.982 to 1.029)	0.651
	ASA ≥3	3.156 (1.376 to 7.236)	0.007
	History of chemotherapy	1.457 (0.839 to 2.530)	0.182
	Prolonged operation (≥4 h)	1.443 (0.843 to2.471)	0.181

GDFT, goal-directed fluid therapy; ASA, American Society of Anesthesiologists.

For postoperative functional recovery (**Table 5**), time of self-urination, bowel function recovery, and oral intake were not significantly different between the two groups. The average length of stay was 13 vs. 14 days in the control group and the GDFT group, respectively, which also did not differ significantly between the two groups [mean difference = −0.3 (−1.7, 1.1), *P* = 0.706]. Considering that approximately 15% of the patients underwent intestinal anastomosis, we did a sensitivity analysis in patients who did not receive bowel resection and anastomosis. Results still showed no significant differences between the two groups (**Appendix Table S1**). Neither did multivariable analyses adjusting for baseline characteristics draw significant differences (**Appendix Tables S3–5**).

**Table 5:** Postoperative recovery.

	Control (N = 129)	GDFT (N = 120)	P-value
*LOS* [*d*ays*, median* (*IQR)*]	13 (11, 16)	14 (11, 17)	0.706^a^
*PLOS* [*d*ays*, median* (*IQR)*]	9 (8, 13)	10 (8, 13)	0.967^a^
*ICU admission (n, %)*	47 (36.4%)	53 (44.2%)	0.214^b^
*Exhaust* d*ay (d*ays*, mean ± SD)*	2.9 ± 0.9	3.0 ± 1.2	0.879^c^
*Defecation* d*ay (d*ays*, mean ± SD)*	4.4 ± 2.1	4.8 ± 2.1	0.149^c^
*Urination* d*ay (d*ays*, mean ± SD)*	4.2 ± 2.5	4.4 ± 4.0	0.556^c^
*Liquid* i*ntake* d*ay (d*ays*, mean ± SD)*	4.5 ± 2.8	4.3 ± 2.5	0.563^c^
*Semi-*liquid intake d*ay (d*ays*, mean ± SD)*	6.5 ± 4.3	6.4 ± 2.9	0.736^c^

LOS, length of stay; PLOS, postoperative length of stay.

a Mann–Whitney U test.

b Chi-square test.

c Student’s t-test.

## Discussion

Intraoperative fluid management has been challenging in patients undergoing gynecologic oncology surgeries. Our study demonstrates that intraoperative goal-directed fluid management benefits these patients in terms of reducing postoperative complications.

Patients undergoing major gynecologic oncology surgery often experience dramatic fluid loss and subsequent massive infusion during perioperative management. Open laparotomy is currently the major surgical technique in cytoreductive surgery as well as comprehensive staging surgery. Gynecologic surgeries of advanced malignancies often require prolonged surgical time (average > 4 h in our study), extensive surgical trauma, and sometimes excision of multiple organs including the gastrointestinal tract, liver, gallbladder, and urinary tract performed by a team of surgical specialists. Significant blood loss due to surgical procedures can be easily foreseen. Another highlighted trait of gynecologic cancer patients is the prominent malignant ascites in around one-third of patients with advanced cancer (6). While some gynecologists choose to perform paracentesis before surgery to relieve patients’ burden, some gynecologists drain the ascites intraoperatively. Therefore, massive fluid loss should be estimated for these patients. Moreover, as these patients often require preoperative bowel preparation and fasting, they are at a higher risk of hypovolemia preoperatively. Therefore, considering drastic intraoperative volume exchange, goal-directed fluid therapy is of clinical significance in this group of patients.

In this study, patients in the GDFT group received significantly less fluid infusion than the control group (15.8 vs. 17.9 ml/kg/h, *P* = 0.013), while fluid loss was similar between the two groups (6.9 vs. 7.1 ml/kg/h, *P* = 0.691). A larger amount of fluid was infused in our group of patients compared with the other types of surgery including laparoscopic gastric bypass surgery (7), colorectal surgery (8), and esophageal surgery (9). Even compared with patients undergoing other open abdominal surgeries (including visceral, vascular, and urology surgeries) receiving goal-directed fluid therapy (10), patients in our study received more fluids intraoperatively (15.8 vs. 10.8 ml/kg/h). Compared with another study conducted by our research team following a similar methodology in major spine surgeries (11), intraoperative fluid transfusion was almost twice the amount (15.8 vs. 8.8 ml/kg/h). Tackling massive blood loss, more than 45% of the patients received blood products of various types in our study, and both crystalloids and colloids were constructively administered for resuscitation. However, blood loss could not fully explain the results as the estimated blood loss was similar between major gynecology oncology surgeries and spine surgeries of around 500 ml on average. This well-demonstrated that fluid loss other than blood, including loss of fluid due to preoperative bowel preparation, invisible fluid loss during prolonged surgery, and loss of ascites, should be specifically considered during fluid management for gynecology oncology patients.

Our study suggested that the implementation of intraoperative goal-directed fluid management in patients undergoing major gynecologic oncology surgeries was associated with reduced risk of postoperative morbidities (OR = 0.421, 95% CI 0.241 to 0.733, *P* = 0.002), especially surgical site infections (SSI). This finding is consistent with multiple clinical trials and meta-analyses in patients undergoing major non-cardiac surgeries (12–14).

As observed in this study, GDFT was significantly associated with a reduced risk of surgical site infections (OR = 0.127, 95% CI 0.003 to 0.971, *P* = 0.037). While the development of SSI involves complex mechanisms and risk factors including diabetes, obesity, cardiovascular diseases, smoking, malignancy, history of radiotherapy, and malnutrition (15), patients in this study with gynecologic malignancy were more susceptible to SSI in the sense of baseline condition. In the setting of open gynecologic surgeries, healing of a long surgical incision might be affected by tissue edema, improper perfusion, and oxygenation of the incision site (16). As GDFT aims to provide adequate perfusion at end organs including the skin, it may benefit wound healing in these patients.

In terms of edema at other sites, a higher incidence of cardiovascular and gastrointestinal complications and lymphocele was observed in the control group, although the difference was not significant. Hypervolemia might be a reason for the poorer postoperative outcome for patients in the control group, causing interstitial edema and organ dysfunction, exacerbating oxygen and metabolite diffusion, and impairing tissue perfusion and tissue architecture. Venous outflow and lymphatic drainage might also be obstructed under the condition of hypervolemia (17). The incidence of pulmonary complications was not different between the two groups, although a previous study discovered that larger fluid infusion was associated with a higher risk of respiratory complications with a dose–effect relationship (13). This might result from our relatively small study population and low incidence of these complications.

Previous studies have drawn conflicting results about whether GDFT benefits postoperative bowel function recovery. Both hypovolemia and hypervolemia can be detrimental to bowel function recovery. One meta-analysis summarized the data of 1,836 patients undergoing gastrointestinal surgery and concluded that GDFT did not reduce the incidence of ileus (18). In this study, GDFT did not show benefit in bowel function recovery, neither reducing the incidence of ileus nor advancing time of postoperative oral intake. In contrast, a matched case–control study in 44 patients undergoing primary debulking gynecological surgery suggested that goal-directed hemodynamic management benefited patients from faster bowel function recovery and shorter hospital stay (19). Although a similar surgical population was studied, the patients included in Dr. Russo’s study had no comorbidities other than tumor load and fewer patients required postoperative intensive care, while the patients in our study suffered more from complex baseline comorbidities. On the other hand, different intraoperative management protocols based on different hemodynamic parameters were followed, resulting in limited generalizability of its result. It was worth noticing that the median volume of total crystalloid infusion in the control group of Dr. Russo’s study was 5,150 ml, almost 70% more than that in the intervention group (2,950 ml), while it was 2,700 ml (control group) vs. 2,500 ml (GDFT group) in our study, which might be an explanation for our different findings. In addition, as multiple factors could influence bowel function recovery, apart from fluid balance, factors including opioid dosage, obesity, previous abdominal surgery, massive blood loss, preoperative albumin, and increased size of incision can all influence bowel motility (20), and intraoperative fluid management might not play a crucial role in the whole postoperative recovery process, especially in a group of patients undergoing major gynecologic surgery who often receive long incision and experience massive blood loss and relatively long surgical time with abundant opioid administration.

For other postoperative recovery indicators, although feeding within the first 24 h is recommended in the 2019 ERAS guideline for gynecologic oncology (2), at the time this trial was performed, early feeding was poorly followed in routine practice in the gynecology ward. Length of stay was also not significantly different between the two groups, partly because patients often stayed in the hospital until the first course of postoperative chemotherapy in our institute. Length of stay was more dependent on the gynecologists’ decision to initiate chemotherapy and discharge afterward, rather than the common recovery standard of leaving the hospital.

The principle of goal-directed fluid therapy is using hemodynamic parameters to guide the adequate use of fluid, inotropes, and vasopressors. The basic physiology of goal-directed fluid therapy involves a complex cardiovascular response (21). It has been estimated that approximately half of the perioperative patients are fluid non-responders with no increase in cardiac output after initial fluid resuscitation (22), while preinduction fluid optimization was not associated with postinduction hypotension (23). This indicates that hypovolemia is not the definite reason for perioperative hemodynamic instability. Cardiac output is greatly influenced by the interaction between stressed and unstressed intravascular volume, and an adequate amount of vasopressor might help fluid shift from unstressed volume into stressed volume and therefore improve cardiovascular function as well as perfusion (21). Under the condition of general anesthesia and mechanical ventilation, GDFT should guide anesthesiologists to apply not only fluids but also vasopressors and inotropic medications according to stroke volume response rather than apparent drop in blood pressure, capturing the time before prominent hemodynamic instability causing hypoperfusion. This also correlates with our result that generally more patients in the GDFT group were administered with vasopressors, especially more continuous infusion of phenylephrine was applied in GDFT patients, which might provide more adequate perfusion with better postoperative outcome. This suggests that both fluid replenishment and application of vasopressors and inotropes are all inseparable contents in a comprehensive GDFT plan.

Our study faces several limitations. The before-and-after chronological design resulted in unbalanced baseline characteristics of the patients. In this study, patients in the GDFT had more baseline comorbidities and higher ASA-PS classification. This might be partly a result of the position as a tertiary center for complicated cases of our medical institution. However, the results still demonstrated a lower incidence of postoperative complications in a group of patients with more severe medical conditions, further indicating an actual benefit of GDFT. The before-and-after design might also bring potential bias due to other possible changes over time (e.g., proficiency in surgical skills); therefore, the results should be interpreted with caution. In addition, the relatively small sample size and single-center study design may affect the generalizability of our results. Comprehensive perioperative ERAS management could be incorporated in future studies.

## Data Availability Statement

All data are available by e-mail request to the corresponding author when required.

## Ethics Statement

The studies involving human participants were reviewed and approved by the Research Ethics Committee of Peking Union Medical College Hospital. The patients/participants provided their written informed consent to participate in this study.

## Author Contributions

JY, LX, and YH contributed to the study conception and design. JY, LC, and LX contributed to data interpretation and preparation of the manuscript. JY and AZ contributed to data acquisition and statistical analysis. All the authors granted final approval of the manuscript.

## Conflict of Interest

The authors declare that the research was conducted in the absence of any commercial or financial relationships that could be construed as a potential conflict of interest.

## Publisher’s Note

All claims expressed in this article are solely those of the authors and do not necessarily represent those of their affiliated organizations, or those of the publisher, the editors and the reviewers. Any product that may be evaluated in this article, or claim that may be made by its manufacturer, is not guaranteed or endorsed by the publisher.
