# Supplementary material for: Goal-Directed Intraoperative Fluid Therapy Benefits Patients Undergoing Major Gynecologic Oncology Surgery: A Controlled Before-and-After Study
Source: Front Oncol. 2022 Apr 6;12:833273. doi: 10.3389/fonc.2022.833273 (PMC9019364; doi:10.3389/fonc.2022.833273)
Supplement: Supplementary file 1 [file Table_1.docx]

Appendix. Supplementary Tables

Table S1 Subgroup analysis of postoperative complications and functional recovery

|  | *Control (N=129)* | *GDFT (N=120)* | *P value* |
| --- | --- | --- | --- |
| ***Patients with one or more complications, n (%)*** | 85 (65.9%) | 63 (52.5%) | 0.032^†^ |
| Standard CRS (N=211) | 71(65.1%) | 50(49.5%) | 0.022^†^ |
| CRS with bowel resection (N=38) | 14(70.0%) | 13(68.4%) | 0.915^†^ |
| ***Length of stay (d, median [IQR])*** | 13 (11,16) | 14 (11,17) | 0.706^††^ |
| Standard CRS (N=211) | 12(11,14) | 13(10,16) | 0.283^††^ |
| CRS with bowel resection (N=38) | 18(15,24) | 17(14,20) | 0.212^††^ |
| ***Exhaust Day (d, mean±SD)*** | 2.9 ± 0.9 | 3.0 ± 1.2 | 0.879^‡^ |
| Standard CRS (N=211) | 2.9±0.8 | 2.8±1.2 | 0.722^‡^ |
| CRS with bowel resection (N=38) | 3.3±1.4 | 3.6±1.0 | 0.340^‡^ |
| ***Liquid Intake Day (d, mean±SD)*** | 4.5 ± 2.8 | 4.3 ± 2.5 | 0.563^‡^ |
| Standard CRS (N=211) | 3.8±1.6 | 3.7±1.7 | 0.228^‡^ |
| CRS with bowel resection (N=38) | 8.4±4.5 | 7.6±3.4 | 0.520^‡^ |

* CRS: cytoreductive surgery

†: Chi-square test; ††Mann-Whitney U test; ‡: Student’s t test.

Table S2 Multivariable logistic regression analysis for postoperative complications

| Model | Variables | Odds ratio (95%CI) | P value |
| --- | --- | --- | --- |
| Crude | GDFT | 0.572 (0.343 to 0.953) | 0.032 |
| Adjusted Model 1 | GDFT | 0.421 (0.241 to 0.733) | 0.002 |
|  | Age | 1.005 (0.982 to 1.029) | 0.651 |
|  | ASA-PS ≥ 3 | 3.156 (1.376 to 7.236) | 0.007 |
|  | History of chemotherapy | 1.457 (0.839 to 2.530) | 0.182 |
|  | Prolonged operation(≥4hrs) | 1.443 (0.843 to2.471) | 0.181 |
| Adjusted Model 2 | GDFT | 0.483 (0.280 to 0.833) | 0.009 |
|  | Age | 1.019 (0.995 to 1.045) | 0.123 |
|  | Hypertension | 1.233 (0.612 to 2.485) | 0.558 |
|  | Diabetes mellitus | 0.351 (0.152 to 0.811) | 0.014 |
|  | Coronary artery disease | 1.663 (0.296 to 9.341) | 0.564 |
|  | Cerebral infarction | 1.908 (0.309 to 11.774) | 0.487 |
|  | History of chemotherapy | 1.238 (0.711 to 2.157) | 0.450 |
|  | Prolonged operation(≥4hrs) | 1.383 (0.809 to 2.364) | 0.237 |

*GDFT: goal-directed fluid therapy; ASA: American Society of Anesthesiologists

Table S3. Multiple linear regression for length of stay

| Factors | Coefficients | 95%CI | P value |
| --- | --- | --- | --- |
| **GDFT management** | **0.208** | **(-1.191, 1.606)** | **0.770** |
| Age | 0.025 | (-0.033, 0.083) | 0.396 |
| ASA-PS≥3 | 0.566 | (-1.354, 2.487) | 0.562 |
| History of chemotherapy | -1.143 | (-2.574, 0.288) | 0.117 |
| History of surgery | 3.561 | (1.948, 5.173) | <0.001 |
| Prolonged surgery | 2.640 | (1.291, 3.989) | <0.001 |

Table S4. Multiple linear regression for liquid intake day

| Factors | Coefficients | 95%CI | P value |
| --- | --- | --- | --- |
| **GDFT management** | **-0.223** | **(-0.860, 0.414)** | **0.491** |
| Age | 0.036 | (0.009, 0.062) | 0.009 |
| ASA-PS≥3 | 0.119 | (-0.757, 0.994) | 0.790 |
| History of chemotherapy | 0.194 | (-0.458, 0.847) | 0.558 |
| History of surgery | 1.839 | (1.108, 2.571) | <0.001 |
| Prolonged surgery | 1.368 | (0.753, 1.982) | <0.001 |

Table S5. Multivariable logistic regression for admission to ICU

| Factors | OR | 95%CI | P value |
| --- | --- | --- | --- |
| **GDFT management** | **1.044** | **(0.577, 1.891)** | **0.886** |
| Age | 1.057 | (1.028, 1.087) | <0.001 |
| ASA-PS≥3 | 2.221 | (0.993, 4.967) | 0.052 |
| History of chemotherapy | 0.917 | (0.496, 1.696) | 0.784 |
| History of surgery | 1.304 | (0.659, 2.583) | 0.446 |
| Prolonged surgery | 3.563 | (2.009, 6.320) | <0.001 |

Table S6 Patients receiving additional resection

|  | *Control (N=129)* | *GDFT (N=120)* |
| --- | --- | --- |
| Bowel resection (n, %) | 20 (15.5%) | 19 (15.8%) |
| Partial hepatectomy (n, %) | 0 (0.0%) | 1 (0.8%) |
| Cholecystectomy (n, %) | 1 (0.8%) | 0 (0.0%) |
| Splenectomy (n, %) | 2 (1.6%) | 1 (0.8%) |
| Partial cystectomy and repair (n, %) | 1 (0.8%) | 3 (2.5%) |
| Partial diaphragm resection and repair (n, %) | 1 (0.8%) | 0 (0.0%) |

Table S7 Postoperative chemotherapy information

|  | *Control* | *GDFT* | *P value* |
| --- | --- | --- | --- |
| Patients who received postoperative chemotherapy during hospital stay (n, %) | 100 (77.5%) | 92 (76.7%) | 0.873^†^ |
| Day of receiving chemotherapy after surgery (d, median [IQR]) | 8 [7, 10] | 8 [7,11] | 0.699^††^ |
| Day of discharge after end of chemotherapy (d, median [IQR]) | 1 [0, 2] | 1 [0, 3] | 0.800^††^ |

†: Chi-square test; ††Mann-Whitney U test;
